# Genitourinary Syndrome of Menopause: A Narrative Review Focusing on Its Effects on the Sexual Health and Quality of Life of Women

**DOI:** 10.7759/cureus.48143

**Published:** 2023-11-02

**Authors:** Vaibhavi B Wasnik, Neema Acharya, Shazia Mohammad

**Affiliations:** 1 Department of Obstetrics and Gynaecology, Jawaharlal Nehru Medical College, Datta Meghe Institute of Higher Education and Research (Deemed to be University), Wardha, IND

**Keywords:** low dose estrogen therapy, vaginal maturation index, sexual health, atrophic vaginitis, vulvovaginal atrophy, genitourinary syndrome of menopause

## Abstract

Genitourinary syndrome of menopause (GSM) is a progressive condition due to a hypoestrogenic state affecting perimenopausal and menopausal women. GSM was previously known as urogenital syndrome, vulvovaginal atrophy, or atrophic vaginitis. The term vulvovaginal atrophy did not encompass the symptoms of the urinary tract like incontinence, urgency, and discomfort, or allude that it is due to a hypoestrogenic state. Although a significant segment of the population is affected by GSM, it is very sparsely studied, detected, and treated. GSM affects the quality of life and sexual health of most menopausal women suffering from it. Only a few healthcare providers ask about the symptoms of GSM and a tiny percentage of women seek consultation for it. This may be because they are either embarrassed or believe it to be a part of the natural process of aging.

As the life expectancy of women has increased in general, the prevalence of GSM has also risen, while it still remains underdiagnosed and untreated. Properly educating women so that they can seek consultation regarding symptoms of GSM, and training healthcare professionals about communicating with the patient, as well as correctly identifying, diagnosing, and managing the patient are all important to overcome this communication barrier. Once we cross the barrier of diagnosing patients with GSM, we still have to manage the patients with tailor-made prescriptions according to the severity of the symptoms and their preferences. While there are various treatment options, the most effective one is low-dose topical estrogen therapy. In this review, we intend to explore the existing knowledge about GSM and its effect on the quality of life and sexual health of women along with the treatment options for managing and reversing the effects of GSM.

## Introduction and background

Genitourinary syndrome of menopause (GSM), previously known as vulvovaginal atrophy, atrophic vaginitis, or urogenital atrophy, constitutes a spectrum of changes in urogenital tissue and its consequences associated with hypoestrogenic states present in, but not limited to, postmenopausal women [1]. It involves changes in the urogenital tissues like labia majora and minora, introitus, vagina and lower urinary tract. GSM symptoms can be classified into (a) genital symptoms like dryness, irritation, burning, itching, and abnormal vaginal discharge, (b) sexual symptoms like lack of lubrication, discomfort, and dyspareunia, and (c) urinary symptoms like urgency, frequency, discomfort while urination, urinary incontinence, and recurrent urinary tract infections [2]. Around 40-54% of postmenopausal women and 15% of premenopausal women are affected by GSM but only a few of them seek consultation. Around 60% of these women have never had a diagnosis of GSM before [3,4]. GSM is also seen in women receiving anti-estrogenic treatment for breast cancer, especially aromatase inhibitors [5].

Many women do not seek consultation for GSM as they are either shy or embarrassed or they believe that it is a normal part of the process of aging and nothing can be done about it. Healthcare providers hesitate to ask questions, especially about sexual dysfunction, or are unaware of the management of GSM. Our concerns about menopause usually start with the presence of hot flashes and ends with vaginal dryness. However, there are many aspects of GSM, including its negative effect on a woman’s sexual health and psychological impact on their self-image and relationships, that affect their quality of life, which are often ignored [6]. According to a study conducted in a tertiary care hospital in Kathmandu, the vaginal symptoms mainly affect sexual health but have little effect on day-to-day activities [7]. An increase in life expectancy means that one-third of a woman's life is spent postmenopause, which makes it important for us to have a better understanding of GSM and other disorders related to low circulating estrogen, in order to provide more comprehensive care for the physical, sexual, and mental well-being of women [8]. Another challenge is the sparsity of studies about the effects of GSM on the well-being of women and their day-to-day lives in the Indian context, and hence further research is required on this topic.

## Review

Methodology

To conduct this narrative review focusing on postmenopausal women suffering from GSM, we searched the database PubMed Central in July 2023 by using keywords such as “Genitourinary Syndrome of Menopause” or “Vulvovaginal atrophy” or “Urogenital atrophy” and “Quality of life” and “Treatment” (((Genitourinary syndrome of menopause) OR Vulvovaginal atrophy) OR urogenital atrophy) AND management) and (((genitourinary Syndrome of menopause) OR Vulvovaginal atrophy) OR urogenital atrophy) AND quality of life). We additionally searched for key references from bibliographies of relevant studies.

**Figure 1:**
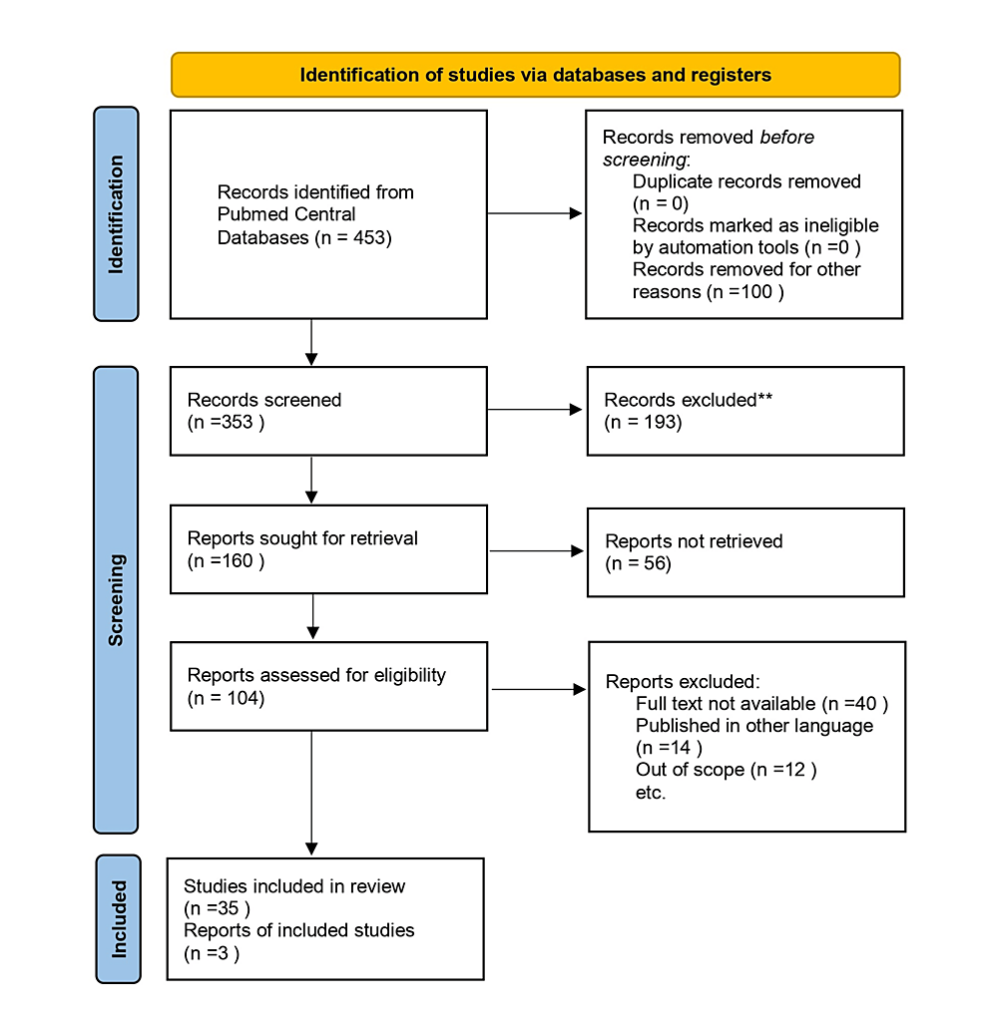
PRISMA flow diagram depicting the selection of studies PRISMA: Preferred Reporting Items for Systematic Reviews and Meta-Analyses

The inclusion criteria included published articles, both original studies and review articles in the English language containing the above-mentioned keywords from 2002 until July 2023. We especially included studies focusing on the quality of life and management of GSM in postmenopausal women. We assessed the relevant studies against the inclusion criteria, based on the abstract and title, followed by analyzing the whole text of the articles. We excluded studies conducted before 2002 and studies in languages other than English. We also excluded studies whose full text was unavailable to us due to resource limitations. We also excluded studies focusing on menopausal changes other than GSM. The details of the database search are depicted in Figure 1*.*

Prevalence

In premenopausal women, the prevalence of genitourinary syndrome is 15%, and it is 40 to 54% in postmenopausal women. About 17% of the population will be over 65 years of age by 2030. Hence, the population of postmenopausal women suffering from hypoestrogenic states will also increase [4]. 

Pathophysiology

The female genital tract and the lower urinary tract embryologically arise from the same structure, namely the urogenital sinus. The external genitalia and the lower urinary tract thus have a higher number of receptors for estrogen [9]. As menopause occurs, there is a decrease in estrogen receptors as well as estrogen. Estrogen is a vasoactive compound; it promotes the vascularity of these structures and thus, by transduction of fluid, lubrication [4]. It also promotes epithelial cell proliferation, mucin production, and collagen turnover. The vaginal rugae, maintained by estrogen, help in distention, expansion, lubrication, and compliance of the vagina [10]. With a hypoestrogenic state, there is thinning of the labia and vulva, a decrease in collagen, elasticity, decreased vascularity, vaginal atrophy, and reduced vaginal discharge. With the loss of elasticity, there is shortening and narrowing of the vagina, causing dyspareunia. There is also atrophy of the bladder and urethra, leading to urinary symptoms like urinary incontinence and increased frequency [2].

As epithelial cell exfoliates, it releases glycogen, which is hydrolyzed to glucose and converted to lactic acid by normal vaginal flora containing lactobacillus, lowering the pH of the vagina. In a hypoestrogenic state, there is a loss of normal flora, increasing the pH of the vagina, thereby predisposing women to recurrent infections [11].

Risk factors

Other than menopause itself, there are several other risk factors associated with the development of GSM. These include bilateral oophorectomy, hypothalamic amenorrhoea, alcohol abuse, ovarian failure, radiation treatment, treatment by antiestrogenic agents in breast cancer, sexual dysfunction, smoking, and other induced hypoestrogenic states not associated with menopause [12].

Clinical features

**Figure 2:**
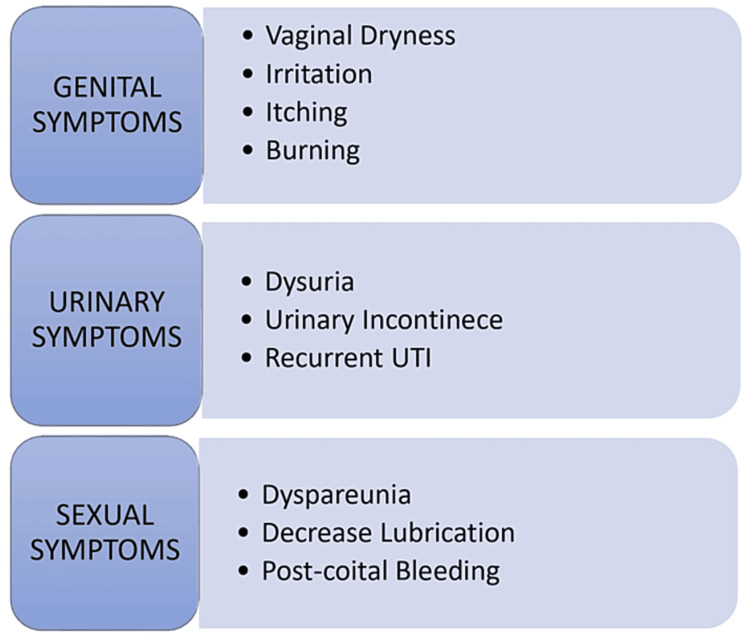
Clinical features of genitourinary syndrome of menopause Image credit: Author (Vaibhavi Wasnik) UTI: urinary tract infection

GSM is a progressive and chronic condition of the external female genitalia and lower urinary tract due to hypoestrogenic states. The diagnosis of GSM is clinical in nature; however, it has very non-specific and mild symptoms, as illustrated in Figure 2* *[13].

As opposed to vasomotor disturbances due to menopause, like hot flashes, night sweats, and sleep disturbances, which reduce with time, GSM symptoms will increase in severity and frequency [7,14]. 

*Vulvovaginal* *Symptoms* *and* *Sexual* *Dysfunction*

Vulvovaginal symptoms include symptoms like vaginal dryness, itching, irritation, and burning sensation and sexual symptoms like decreased lubrication, dyspareunia and bleeding after intercourse and sexual dysfunction. If not effectively managed, GSM can be complicated by uterine prolapse and cystocele [15]. These symptoms are due to vaginal atrophy, as well as decreased lubrication due to decreased circulating estrogen. According to an international survey, VIVA, conducted to study vaginal atrophy, about 45% of postmenopausal women have reported experiencing the above-mentioned signs and symptoms. About 75% of these women reported that these symptoms have negatively affected their life [16].

It is believed that vulvovaginal atrophy and sexual dysfunction have associations with each other. According to a study conducted by Levine et al., women with sexual dysfunction are 3.84 times more likely to develop symptoms of vulvovaginal atrophy. Similarly, women with vulvovaginal symptoms of GSM are more likely to experience sexual dysfunction due to dyspareunia, decreased lubrication, and pain during and bleeding after intercourse [17].

*Lower* *Urinary* *Tract* *Symptoms*

As both the lower urinary tract and female genitalia share the same embryonic origin, they both have a higher number of receptors for estrogen. Due to the hypoestrogenic state, there are atrophic changes in the urogenital tract leading to symptoms like urinary urgency, higher frequency, urinary incontinence, and recurrent urinary tract infections. Urinary incontinence is the main problem during menopause; about 50% of postmenopausal women experience it, stress incontinence being the most common among them [18]. Urinary incontinence is one of the major symptoms of GSM, negatively affecting sexual function, day-to-day activities, and quality of life of these women [19].

The prevalence of UTI is higher in postmenopausal women, especially owing to the loss of normal flora of the vulvovaginal area consisting of lactobacillus responsible for lowering the pH and providing protection against urinary tract infections. Healthcare providers should be aware of the association between GSM and UTI to prevent inconsiderate use of antibiotics [17].

Genitourinary syndrome of menopause: sexual health and quality of life

Sexual health plays an important role in emotional well-being, self-image, and the quality of life in an adult woman regardless of age. There is social stigma present around women and their sexuality, leading to either embarrassment to discuss these issues or misapprehensions about conditions like GSM, which adversely affects their sexual health. The changes, symptoms, and signs in GSM like dyspareunia, post-coital bleeding, reduced lubrication, and urinary incontinence can negatively affect sexual function, interpersonal relationships, and quality of life among postmenopausal women [20]. GSM, sexual dysfunction, and quality of life are all associated with each other. Various studies and surveys have examined this relationship, such as the GENISSE study. The objective of the GENISSE study was to determine the prevalence and impact of GSM on quality of life. Accordingly, it showed that GSM had little effect on the day-to-day functioning of these women. However, it had an effect on the women’s self-perception and also moderately affected sexual functioning in women [3].

Another survey, named CLOSER, was specifically designed to study the effect of GSM on sexual functioning and relationships. It was found that vaginal discomfort due to GSM has negatively affected relationships in about 58% of women [21]. A study by Kingsberg et al., named REVIVE, found that GSM affected sleep in 24% of women and sexual functioning in 59% of women [22].

Diagnosis and investigation of GSM

The diagnosis of GSM is mainly clinical. History and pelvic examination are sufficient to establish a diagnosis. On pelvic examination, various findings like labial atrophy and such are found, which are summarized in the figure below. Other tests can include urinalysis and acid balance tests of vaginal fluids [8].

Another parameter that is rarely used practically is the vaginal health index (VHI). It is used to determine the atrophy of the genitourinary tract. It includes five criteria, namely (1) vaginal secretions, (2) vaginal pH, (3) vaginal elasticity, (4) vaginal petechiae, and (5) vaginal dryness. The lowest score here is 5 and the highest is 25. A score <15 indicates the presence of vaginal atrophy [23].

Another tool is the vaginal maturation index (VMI), which is based on vaginal cytology. The vaginal cytology in GSM shows a preponderance of parabasal cells and fewer number of intermediate and superficial cells. As the vagina becomes atrophied under a hypoestrogenic state, the immature parabasal cells are unable to mature into intermediate and superficial cells. If the percentage of superficial cells is less than 5%, it indicates vaginal atrophy. VMI is not used for clinical diagnosis of GSM but is useful for research purposes [24].

Treatment of GSM

GSM is a chronic and progressive condition; it tends to worsen as time passes, and hence its management is important. The main aim of the treatment is to manage the symptoms. Lifestyle modifications like cessation of smoking are important. This is mainly due to the fact that smoking promotes the metabolism of estrogen and reduces its bioavailability. Another risk factor of GSM is obesity and hence weight loss and physical exercise should be encouraged in these patients [25].

Local Non-hormonal Therapy

Vaginal moisturizers: vaginal dryness is the most prominent symptom of GSM. Vaginal moisturizers are used for relieving vaginal dryness, itching, burning and to reduce dyspareunia during intercourse. Vaginal moisturizers are also preferred in women who only experience vulvovaginal symptoms of mild severity and not urinary symptoms. The parameters of VHI are all positively affected and the VHI score is increased [25]. The long-term use of vaginal moisturizer also increases the vaginal elasticity [26]. Comparative studies have shown that the effectiveness of polycarbophil-containing vaginal moisturizers is similar to that of local estrogen therapy [27]. Many vaginal moisturizers contain hyaluronic acid. Hyaluronic acid has the ability to trap substantial amount of water molecules, which is then slowly released, thereby maintaining the water balance. Hyaluronic acid also plays an important role in cell repair and maintaining tissue integrity. Hyaluronic acid-based gel has higher safety profiles as compared to local estrogen therapy [28].

Vaginal lubricants: lubricants can be either water-based, silicone-based, or oil-based. Water-based variants are most commonly used. Oil-based variants can promote the colonization of candida species and are thus not preferred. Another disadvantage of oil-based lubricant is that it causes latex condom breakdown, leading to an increased risk of sexually transmitted infections [29]. It is a temporary method for relief of vaginal dryness and hence can be used before intercourse to avoid dyspareunia [25]. If the osmolality and the pH of the vaginal lubricants differ from the normal vaginal pH and osmolality, it can predispose women to bacterial vaginosis. Higher osmolality of the lubricants also directly relates to mucosal irritation and contact dermatitis. It has been recommended by the WHO to use lubricants with an osmolality of less than 350 mOsm/kg with a pH of 4.5 [29]. Vaginal lubricants can be used as an adjunct to vaginal moisturizers as a first-line therapy for mild to moderate symptoms of GSM [30].

*Estrogen* *Therapy* (*ET*)

In case of moderate to severe GSM or if other treatment modalities do not give satisfactory results, low-dose local topical estrogen therapy is preferred. It is one of the most effective therapeutic options for GSM and is the most commonly prescribed. Topical estrogen is absorbed through the vaginal mucosa, improving the blood supply, lowering the pH, thickening the vaginal epithelium, and improving vaginal lubrication [31]. There are various formulations of estrogen-like vaginal rings, creams, gel, pessaries, and tablets that are being used. The severity of symptoms as well as the patient’s preferences are factored in when deciding among the different preparations. It has been found in a Cochrane 2006 review that all the preparations are equally effective in managing GSM. The adverse effects associated with the use of any of the formulations of topical estrogen therapy are vaginal itching, irritation, and discharge leading to noncompliance among patients [32]. Other adverse effects like endometrial hyperplasia were seen with high-dose (25 μg) administration of estradiol. While prescribing topical estrogen therapy, the lowest effective dosage should be considered. As there is no risk of endometrial hyperplasia with a low dose of estrogen, the use of progesterone as well as endometrial monitoring is not indicated. In patients with hormone-sensitive cancers like breast, endometrial, ovarian, or cervical adenocarcinoma, estrogen therapy is usually avoided [33].

Systemic hormonal therapy is preferred when both vasomotor symptoms, as well as vulvovaginal atrophy, are present simultaneously. Systemic ET is given alongside progesterone, to reduce the risk of endometrial hyperplasia. In cases of women who have undergone hysterectomy, estrogen can be administered alone [34]. However, clinicians need to do a risk analysis given the several side effects of systemic estrogen therapy. Common but less severe side effects of systemic ET include breast pain, headache, and irregular vaginal bleeding. Stroke, breast cancer, ovarian cancer, and fibroids are some of the more serious but less prevalent side effects [35].

*Vaginal* *Dehydroepiandrosterone* (*DHEA*)

DHEA is a precursor to estrogen; its local application causes increased conversion to estrogen without any effect on the endometrium. Daily application of vaginal DHEA has been shown to improve symptoms of GSM [25]. According to original research by Labrie et al., daily intravaginal application of DHEA improved VMI, vaginal pH, and dyspareunia. It is preferred in patients who are contraindicated to use estrogen therapy, such as breast cancer survivors using tamoxifen [36].

Ospemifene

Ospemifene is an oral preparation of a selective estrogen receptor modulator (SERM), which selectively acts on the vaginal tissue. It improves VMI and reduces the pH of the vagina and reduces the incidence of dyspareunia [25]. Other preparations of SERM include tamoxifen, raloxifene, and lasofoxifene; however, these are not approved by the FDA. Ospemifene is contraindicated in patients with a high risk of VTE. Patients usually prefer oral ospemifene over topical ET and it is associated with higher patient compliance. Due to a lack of studies on the use of ospemifene in patients with breast cancer, it is usually not prescribed [37].

Laser

Laser is indicated in the following three situations: (a) failure of other therapeutic options like vaginal moisturizers and topical and systemic ET, (b) contraindications in the use of topical or systemic estrogen-like breast cancer survivors, (c) unwillingness to use other treatment options. Laser acts by noninvasively increasing collagen production and connective tissue remodeling. Depending on the energy source, lasers that can be used are either a CO_2_ laser or erbium-YAG laser; however, the former has been the most commonly used type in the treatment of GSM (15). The use of laser has shown improvement in symptoms of GSM including vaginal, urinary as well sexual functioning, and it is well tolerated. There is also increased acidity in the pH levels. There is symptomatic relief as well as improvement in vaginal cytology. However, a few things still need to be studied when it comes to the use of laser, like how long the effect of treatment lasts and what are the long-term effects of using laser for GSM [38]. The findings of the articles reviewed are summarized in Table 1.

**Table 1:** Summary of the findings of the studies included in the review NAMS: North American Menopausal Society; ISSWSH: International Society for the Study of Women's Sexual Health; GSM: genitourinary syndrome of menopause; UI: urinary incontinence; VMI: vaginal maturation index; VHI: vaginal health index; ET: estrogen therapy; QoL: quality of life; HCP: healthcare provider; VA: vaginal atrophy

Author	Year	Findings
Portman and Gass [1]	2014	This review article states that vulvovaginal atrophy, by consensus of NAMS and ISSWSH, is now termed genitourinary syndrome of menopause, which incorporates signs and symptoms associated with the hypoestrogenic state including genital, sexual, and urinary symptoms
Nappi et al. [2]	2019	This study states that GSM is an early predictor for poor general health. The same is true for vasomotor symptoms. Healthcare practitioners should be proactive in diagnosing and managing GSM for the overall well-being of postmenopausal women
Moral et al. [3]	2018	This study found that the prevalence of GSM in Spanish women is about 70%, with the most prominent clinical features being vaginal dryness, UI, and dyspareunia. It has a low to moderate impact on the quality of life of the women affected
Gandhi et al. [4]	2016	According to this study, GSM is a prevalent condition in postmenopausal women, which is often undetected. Early diagnosis and custom-made prescriptions positively affect the quality of life of women and also prevent it from worsening
Cook et al. [5]	2017	This study concluded that breast cancer survivors who are on aromatase inhibitor therapy often experience GSM symptoms and sexual dysfunction, which negatively affects their quality of life. GSM assessment should be integrated into the care for breast cancer survivors
Peters [6]	2021	According to this study, diagnosis of GSM includes patient history and external genitalia examination. The findings on external genitalia examination include pale introitus, loss of vaginal rugae and elasticity, and increase in pH. Various treatment options include vaginal lubricants, moisturizers, and hormonal therapy
Ojha et al. [7]	2022	A study conducted in a tertiary care center in Nepal shows that 78.4% of postmenopausal women visiting the hospital were diagnosed with GSM. It negatively affected the quality of life of these women, mainly their sexual health
Sarmento et al. [8]	2021	According to this study, vaginal assessment for GSM is done using VMI, vaginal pH, and VHI
Angelou et al. [9]	2020	This study states that local ET is considered the gold standard for GSM. Other first-line therapies include moisturizers, lubricants, and lifestyle changes. Selective estrogen receptor modulators and laser therapy are further evolving as an option for the management of GSM
Briggs [10]	2020	According to this study, sexual health is affected by loss of lubrication, shrinkage of introitus, and loss of elasticity in GSM. Vaginal symptoms and urinary symptoms also affect the quality of life of women. Assessment is done by vaginal pH and vaginal smear to look for vaginal maturation index
North American Menopause Society [11]	2007	According to NAMS, low-dose topical ET is recommended for vaginal atrophy and is given as long as the symptoms persist. Progesterone is not recommended in low-dose ET. In non-hormonal cancer patients as well as patients with no history of cancer, low-dose estrogen therapy is preferred. In hormone-dependent cancers, oncologists should be consulted
Mac Bride et al. [12]	2010	According to this study, the clinical findings of GSM include pale and dry vaginal mucosa and petechiae, loss of elasticity and rugae within the vagina. An increase in vaginal pH is suggestive of GSM. The prevalence of GSM is increased in patients undergoing breast cancer treatments; however, low-dose estrogen therapy use in these patients is disputed
Davila et al. [13]	2003	This study found that the physical signs of GSM weakly correlated with the appearance of symptoms. Even though urogenital atrophy occurs in most postmenopausal women, many of them do not develop symptoms. Hence symptoms should not be the only key factor in deciding whether local ET therapy should be started or not
Palma et al. [14]	2016	In this study, around 935 females of an average age of 59 underwent routine gynecological examinations. Among these, 79% were diagnosed with GSM. Physical signs included mucous membrane dryness and paleness, loss of vaginal rugae, and petechiae. Only 30% of these women were previously diagnosed with GSM with many of them on local ET
Kim et al. [15]	2015	This study points out that vaginal atrophy and atrophic vaginitis were all insufficient terms to describe GSM as they do not encompass all symptoms of GSM including sexual dysfunction, urinary symptoms, and vaginal symptoms. It is important to educate women regarding this and manage the symptoms accordingly
Nappi and Kokot-Kierep [16]	2012	In this study, an online survey was conducted to estimate the understanding of vaginal atrophy in women. Around 41% of these participants suffered from vaginal symptoms; 63% of women did not know about vaginal atrophy and 46% of the participants did not know about local ET. This study concluded that postmenopausal women had a poor understanding of vaginal atrophy
Levine et al. [17]	2008	This study was done to find the association between vaginal atrophy and sexual dysfunction among postmenopausal women who are sexually active. It concluded that the likelihood of vaginal atrophy was 3.84 times greater in women with sexual dysfunction
Robinson and Cardozo [18]	2003	According to this study, estrogen has physiological effects on the lower urinary tract. Hence it can be used in irritative lower urinary tract symptoms as well as to reverse the effects of urogenital atrophy due to hypoestrogenic states
Felippe et al. [19]	2017	This study was performed to find the relationship between UI and women's sexual health. It concluded that women with UI have a higher prevalence of sexual dysfunction
Thornton et al. [20]	2015	This study suggests that sexual function decreases with age, which negatively affects QoL. HCPs should therefore be forthcoming in asking menopausal and postmenopausal women about sexual dysfunction and listen to their concerns about the same
Nappi et al. [21]	2013	This study involved a survey to study the effect of VA on sexual health and relationships. It concluded that VA has negatively affected the physical and emotional well-being of women and their partners
Kinsberg et al. [22]	2013	This study conducted on postmenopausal women concluded that around 38% of participants experienced GSM symptoms; 56% of participants had discussed this with their HCP and 44% of them were on some sort of treatment. Initiatives to improve HCP and patient communication are the key to early diagnosis and management
Henry-Okafor et al. [23]	2021	According to this review article, the reason for GSM being underdiagnosed is women not wanting to discuss this with their HCP. Thus HCPs should be forthcoming in screening postmenopausal women coming to them as well as prescribing therapy suitable for their individual needs
Palacios [24]	2019	According to the study, objective methods of diagnosing GSM include VMI and vaginal pH. These along with clinical history should guide the treatment of GSM
Palacios et al. [25]	2015	The authors recommend symptomatic relief as the main aim of treatment. This includes lubricants and moisturizers, systemic and topical ET, and newer modalities like SERM and laser
Brown and Bachman [26]	2005	According to the authors, even with newer modalities like SERM and laser therapy, low-dose topical ET remains the mainstay of treatment of GSM with minimal systemic absorption
Nachtigall [27]	1994	This study was done to compare the use of vaginal moisturizers with topical estrogen therapy. It concluded that vaginal moisturizer is as effective as local ET for increasing vaginal moisture and decreasing the symptoms
Nappi et al. [28]	2022	This study states that hyaluronic acid is a valid and safe prophylactic option in hormonal cancer patients as well as those unwilling to use ET
Sinha and Ewies [29]	2013	According to this study, lubricants provide no long-term effect on the GSM manifestations; however, it is safe and efficient to use to prevent dyspareunia during sexual intercourse
Sarmento et al. [30]	2021	This study states that in breast cancer survivors, the use of ET is disputed and hence nonhormonal options such as moisturizers and lubricants are to be used
The NAMS 2020 GSM Position Statement Editorial Panel [31]	2020	According to this study, individual patient preference, safety, and other comorbidities should direct the use of various treatment therapies for GSM
Suckling et al. [32]	2006	As per this study, estrogen therapy can be either systemic in the form of tablets and injections or topical in the form of pessaries, rings, and creams
Sturdee and Panay [33]	2010	According to this study, local estrogen is safe and efficient and thus preferred. However, if the patient is unwilling to use hormonal therapy, moisturizers and lubricants are used
Parish and Gillespie [34]	2017	According to this article, oral estrogen is used for vasomotor symptoms due to menopause as well as to improve VMI. However, it can increase the risk of stroke and thromboembolism and risk and benefit should be evaluated before prescribing
Kagan et al. [35]	2019	According to the authors, even though many treatment options are available for GSM, most patients are dissatisfied with the response. Hence better communication between HCPs can help ensure better management
Labrie et al. [36]	2008	According to this study, intravaginal application of DHEA helps in alleviating the symptoms of GSM and can be used safely in breast cancer patients
The North American Menopause Society [37]	2013	According to this study, symptomatic management of GSM can positively impact the QoL of women. Depending on symptom severity as well as the patient's need, various management modalities can be used
Benini et al. [38]	2022	According to the authors, laser therapy is emerging as an effective and safe option for the management of GSM. However, the long-term effects of laser therapy have not been studied

## Conclusions

While the prevalence of GSM has increased with the increase in the population of menopausal women and their life expectancy, it is still underdiagnosed and untreated. The symptoms of GSM are bothersome and affect the quality of life of these women as well as their sexual functioning and social well-being. Women should be educated about the symptomatology of GSM so that they can seek consultation when required; also, healthcare workers should be trained to correctly identify these symptoms and manage them. New tools should be developed to screen perimenopausal and menopausal women for the symptoms of GSM.

The treatment should be decided on the basis of the severity of symptoms as well as the personal preferences of the patient. Topical estrogen is the most effective among all the treatment options. One of the major issues in the management of GSM is the patient's non-compliance with management strategies. Minimally invasive vaginal laser is an emerging method for the management of GSM. Laser has been shown to be effective in treating GSM, even on long-term follow-ups. However, due to the small size of the studies, we are unable to provide information regarding the patient characteristics associated with a positive response to lasers. There is a need for more studies in order to describe the predictive factors that can point toward a positive or negative response to laser treatment. This can help to individualize treatment modalities for these patients.
